# Theory Meets Practice for Immune Checkpoint Blockade in Small-Cell Lung Cancer

**DOI:** 10.1200/JCO.2016.69.0040

**Published:** 2016-09-06

**Authors:** Jonathan W. Riess, Primo N. Lara, David R. Gandara

**Affiliations:** Jonathan W. Riess, Primo N. Lara Jr, and David R. Gandara, University of California Davis Comprehensive Cancer Center, Sacramento, CA

Extensive-stage small-cell lung cancer (ES-SCLC) is an aggressive disease characterized by high initial response rates to first-line platinum-based chemotherapy followed inevitably by relapse, poor response to subsequent systemic treatment, and ultimately death. Long-term survival prospects for ES-SCLC are dismal, with an estimated 2-year overall survival (OS) rate of less than 5%. Recent advances in the development and regulatory approval of several new active agents against advanced non–small-cell lung cancer (NSCLC) contrast sharply with the lack of progress in the systemic treatment of ES-SCLC, where survival outcomes have changed minimally over a quarter century.^1,2^ In fact, the last new drug approval for ES-SCLC (ie, topotecan) occurred almost 20 years ago; meanwhile, 16 new therapies for NSCLC were approved over the same time period (eight targeted therapies, four chemotherapies, two antiangiogenic agents, and two programmed death-1 [PD-1] immune checkpoint inhibitors).

In theory, if any class of drugs were to alter the natural history of ES-SCLC and improve survival, it would be immune checkpoint inhibitors (anti–cytotoxic T-cell lymphocyte-4 [anti–CTLA-4] and anti–PD-1 or anti–programmed death ligand 1 [PD-L1] antibodies). Immune checkpoint blockade is reportedly more active in cancers with hypermutated phenotypes, such as malignant melanoma, NSCLC, bladder cancer, and microsatellite instability–high tumors. The postulated mechanism is that higher neoantigen burden and mutational load render these tumors more immunogenic, with reawakened pre-existent antitumor CD8+ cytotoxic T-cell responses, when exposed to immune checkpoint blockade.^3^ It is thought that high tumoral mutational burden (and thus sensitivity to immunotherapy) corresponds in part to the degree or nature of prior carcinogen exposure. Indeed, smoking-associated NSCLC seems to derive more benefit from checkpoint-targeted immunotherapies than lung cancers in never-smoking patients.^4^ Because lung cancer with small-cell histology has the strongest association with tobacco carcinogenesis and harbors a high frequency of somatic mutations, one would posit that SCLC would preferentially benefit from immune checkpoint blockade.^5,6^ Furthermore, it has been hypothesized that cytotoxic chemotherapy could enhance the expression of tumoral neoantigens, thus priming the tumor for response to checkpoint inhibitor therapy. In fact, in the initial phase II trials of ipilimumab plus chemotherapy in either SCLC or NSCLC, modest improvements in immune-related progression-free survival—based on criteria that accounted for tumor shrinkage in the face of new lesions—were seen when ipilimumab was administered concurrently with chemotherapy in later cycles rather than immediately in the first cycle.^7,8^

Against this background, Reck et al^9^ conducted a large placebo-controlled clinical trial in ES-SCLC in which 1,132 patients were randomly assigned to receive either etoposide and platinum (cisplatin or carboplatin) for four cycles alone or together with the anti–CTLA-4 antibody ipilimumab. Disappointingly, the trial was negative; the primary end point of OS in patients who received at least one dose of ipilimumab was not improved (hazard ratio, 0.94; 95% CI, 0.81 to 1.09).

The phased strategy of delivering two initial cycles of etoposide and platinum without ipilimumab is reasonable given the theoretic considerations we have described for increasing expression of immunogenic neoantigens. Besides, from a practical standpoint, the need for cytoreduction in patients often experiencing symptoms of rapidly growing SCLC is paramount; the high anticipated response rates to initial etoposide and platinum would provide an opportunity to palliate symptoms and enrich the patient population for those more likely to benefit from and tolerate subsequent ipilimumab.^7,8^

Why was this large and well-conducted trial negative? Considerations intrinsic to ES-SCLC likely contributed to the failure of ipilimumab combined with etoposide and platinum to improve outcomes. In this disease, rapid tumor growth with corresponding symptomatic disease and performance status decline can lead to patient drop off as a result of poor drug tolerability or disease progression. In fact, the primary end point in this study was altered from OS in the intent-to-treat population to OS among patients who received at least one dose of study drug commencing at cycle three. As reported by Reck et al,^9^ approximately 15% of randomly assigned patients did not receive the study drug. Only approximately 13% of those randomly assigned to receive ipilimumab lived long enough without progression or toxicity to receive it as maintenance. In other ES-SCLC studies, even when biomarker-driven approaches for immune checkpoint blockade have been used, excessive patient dropout has limited generalizability of clinical outcomes. For example, in KEYNOTE 028, only 24 (16%) of 147 patients with SCLC screened for PD-L1 expression actually received pembrolizumab, although 29% (42 of 147) were PD-L1 positive. Nevertheless, this therapy produced a response rate of 29%, impressive for previously treated ES-SCLC.^10^

Additional potential explanations can be derived from the experience in metastatic melanoma, where it has been reported that cytotoxic exposure before CTLA-4 blockade induces mostly subclonal mutations rather than clonal mutations.^3^ Such subclonal mutations may be insufficient to drive an immune response robust enough to improve survival end points. Perhaps priming doses of chemotherapy in ES-SCLC are unable to generate the appropriate level of neoantigen expression, or perhaps the so-called correct neoantigens are not sufficiently expressed to drive functional immunogenicity. Moreover, as an anti–CTLA-4 targeted agent, ipilimumab may not be the best immunotherapeutic agent to use after chemotherapy, because mechanistically its effect on cytotoxic T cells should occur during the priming phase. Anti–PD-1 or anti–PD-L1 antibodies that act locally in the tumor microenvironment during the effector phase may be more clinically relevant in this context than anti–CTLA-4 antibodies that act peripherally at the time of initial response to antigen.^11^ Indeed, promising overall response rates in trials combining platinum-based chemotherapy with PD-1 antibodies in NSCLC have been reported, although increased toxicity is a major concern; for example, a grade 3 and 4 adverse event rate of 45% and pneumonitis rate of 7%, resulting in discontinuation of study treatment in 21% of patients, were recently reported in a phase I study combining platinum-based chemotherapy and nivolumab.^12^ Maintenance trials with PD-1 antibodies in SCLC after initial cytoreduction with etoposide and platinum are under way and may represent a more tolerable strategy in the population of patients with SCLC, which often has compromised performance status resulting from medical comorbidities and tumor burden.

Rather than priming with cytotoxic chemotherapy, combined CTLA-4 and PD-1 or PD-L1 blockade in SCLC may represent an encouraging alternative combination strategy, although increased toxicity, including risk of paraneoplastic syndromes, which are already more frequent with small-cell histology, remains a major concern. The nonoverlapping mechanisms of action of CTLA-4 and PD-1 blockade are best demonstrated by recent clinical trials reporting the combined effects of agents targeting these two pathways. In the recently published phase I/II CheckMate 032 study, durable responses to nivolumab and ipilimumab were observed, prompting a randomized phase III trial.^13^

Finally, the trial by Reck et al^9^ failed to improve outcomes in part because it did not attempt to enrich for patients who may have preferentially benefited from such a therapeutic strategy. On the basis of early results with immune checkpoint blockade in SCLC, it is likely that only a small subset of patients benefits from these drugs. Thus, continued companion biomarker development and validation to identify those patients likely to respond to immunotherapy are critical. However, tumor samples in ES-SCLC are often scant and inadequate; obtaining adequate tissue in a timely fashion to appropriately assess the tumor and immune microenvironment can be challenging. With a fast-growing cancer like SCLC, there is also a need to identify and exclude patients whose disease will progress too rapidly for potential benefit from immune checkpoint blockade.

How do we put the study by Reck et al^9^ into perspective with other checkpoint immunotherapy trials in lung cancer? A similarly designed phase III trial using first-line carboplatin and paclitaxel with ipilimumab in squamous histology lung cancer is ongoing. Even if positive, it will need to be interpreted within the context of the current widespread use of approved anti–PD-1 agents in squamous cell lung cancer. Understanding the influence of sequencing of prior ipilimumab on clinical outcomes of subsequent PD-1 blockade related to changes in the tumor and immune microenvironment will be important if a meaningful improvement in survival is achieved. These results will also need to be interpreted within the context of the OS benefit recently announced for first-line pembrolizumab in patients with stage IV NSCLC harboring high PD-L1 expression (KEYNOTE 024).

In summary, Reck et al^9^ are to be congratulated for completing, to our knowledge, the largest SCLC trial to date and the first phase III randomized trial with immune checkpoint blockade in SCLC. Although overall survival was not improved by adding ipilimumab to chemotherapy in this trial, recent data suggest that immune checkpoint blockade with dual CTLA-4 and PD-1 inhibition may be a more effective strategy in SCLC.^13^ Assuming toxicity issues are adequately addressed, combined immune checkpoint blockade strategies may be more likely to break the quarter-century drought of new therapies in ES-SCLC.
